# Treatment of Gold Filings

**Published:** 1859-07

**Authors:** L. C. Whiting


### TREATMENT OF GOLD FILINGS.

BY L. C. WHITING.

I HAVE been reading Dr. Geo. Watt's interesting article, in the last number of the *Register*, on the management of gold alloys, and like it very much. What dentists want the most, is not how to make fine gold, but the easiest way of working their filings and old gold plates into gold that can be used for plates or used up in their practice to the best advantage. I have had some practice in refining gold, for the last twelve years, and have tried every plan that the best workmen have recommended, and have finally come to the following, as the best for my purpose:

When my filings have accumulated sufficiently, pass them through a fine sieve to get out all the small pieces of plate that are malleable; do not mix them with the filings, until the filings are melted and in working order. Pass a magnet through the filings, to get out all the iron and steel that may have worked in from your file or otherwise. Add all the old backings of teeth and pieces of plate that have any solder on them; put it into a good-sized crucible, with as much pulverized saltpeter in bulk as there is of the gold; cover it with a smaller crucible and heat up slowly until well melted. As soon as it is all melted, set it off and let it cool in the crucible. Break the crucible, and you will find a button of soft malleable gold, which can be remelted with other gold, or, what is better, used for clasps and backings to teeth. If it does not work well the first time it is melted, and will hold together to roll, pass it through the rollers and make it *very thin*, cut it up with the shears and treat it again the same as the filings. This method has worked much better with me, than the old method of adding saltpeter after it is melted.

Don't allow it to stand over the fire long after it is melted. Never heat very hot or for a long time.

Never use any borax or flux of any kind in melting gold that works well; or at least, what you do use, rub on the crucible to keep the gold from sticking when it is poured. Good gold plate can be made by adding one cent's worth of silver coin to a dollar's worth of gold coin.

